# The Doctor and Increased Production

**Published:** 1919-10-04

**Authors:** 


### The Hospital

The Workers' Newspaper of Administrative Medicine and Institutional Life, Administration, National Insurance and Health.

No. 1739, Vol. LXVII. SATURDAY, OCTOBER 4, 1919. PRICE TWOPENCE

### THE DOCTOR AND INCREASED PRODUCTION.

This is an age of reconstruction, and the keynote to reconstruction is increased production. Every paper that is published, every speech that is made, every review that we pick up, impresses this upon us. Reconstruction consists of two great parts, destruction and construction; there are many who have not yet realised this, and who have begun very vigorously to try to pull down ancient edifices which they find are not ideal without any thought of what they will put in their places. More clear-thinking persons have gone further, and realised that it is useless to pull down unless you have already built something new and improved to take the place of what you intend to remove. They vary greatly in the style, the permanence, and the cost of the new structures that they wish to bring into our civilisation; but they are all agreed that the way to get the raw materials is by increased production.

By increased production, we mean that we must get out of our Mother British earth a greater quantity of her wealth in order that we may buy again from overseas those necessities and reasonable luxuries which she cannot provide for us. And as she gives us only a certain amount of agricultural produce and of the products of our mines, it looks at first sight as though all the nation outside these branches must settle down and do nothing more than they did before, until the workers in these industries have pulled us out of our impending bankruptcy. This is a plausible train of thought, which we believe has unconsciously entered the mind of many people who are now starting upon a period of intended relaxation after the strenuous life of the last five years. We cannot think that it is a correct one, we cannot believe that certain classes who have borne their share in that expenditure of national energy which has won the war have now inherited the duty of restoring the energy which we have expended, while the rest of the nation return to the relative idleness of the pre-war period. We believe that the wealth of the nation depends upon the amount of good work that every man and woman does, and that increased production means an increase of work done by everyone in whatever sphere of life they may be.

In our own profession the contention is that an increase of work upon our own part will lead to an increase of the nation's health. If we can limit the amount of time off-duty from the casual sickness of men and women generally fit, if we can raise the health of those who are usually of a low standard, we shall enable each of these classes of people to put in more time at their work and to do better work. If by preventive measures we eliminate epidemics, everyone will admit that we increase production; but it is less often recognised that we do the same by raising the health of individuals so that they enjoy the work that beforetime was a drudgery, or get a smile out of a life that once was wholly black.

We do not mean by doing more work that medical men or others employed in the art of healing should take on a large number of cases, or should try to extend their spheres of activity. We believe that more good would be done by each one of us trying to do better the work that is already in hand. We do not even believe that an increased number of visits of doctor to patients, or *vice versa*, is necessary. But we do believe that a very material improvement of the health of the nation would result from every one of us taking greater care over our examination of our patients, taking greater thought over the diagnoses that we make, observing more carefully the results of our treatment and advice, and criticising ourselves more frequently to assure ourselves that we were doing our best, rather than allowing that feeling of self-satisfaction to arise which is too common in the older members of our profession. To this we would add the need of more self-education throughout life. This can be got by reading, by discourse, and by thought; in our opinion too great a stress is laid upon the first of these and too little upon the second and third as factors of self-education. There must be few of us who are not in touch with men and women who have had experiences during these five years that we have missed and from whom therefore we have something to learn; few, again, who could not apply to some problem that meets them in civil life the lessons learned from a similar problem which has been faced and solved during their military career.

If any further stimulus is necessary we would suggest that the habit of taking a post-graduate course should be acquired by us in the way it has been in America. It is hard to get away from a busy practice, harder still to go away from one that is in the making; but when the wrench has been made, the week or fortnight will well repay the effort made

There is a way in which we believe that our profession will share with all the educated classes in increasing production by increased work. It is by the force of example. The enormous power of this must have become known to many as a result of their military experience. In a platoon or company the educated man who strove with all his energy at work that was new or distasteful to him, increased the total output by an amount that was quite impossible to his uneducated comrade. In a battalion or other unit the criterion of the amount of work done was the amount which the commanding officer did himself. The educated classes are, and must always be, the true leaders of the country. It is up to us as an educated profession to set an example of good work and increased work to our less cultivated countrymen.

